# Using LDpred2 to adapt polygenic risk score techniques for methylation score creation

**DOI:** 10.1186/s13104-025-07222-2

**Published:** 2025-04-23

**Authors:** Kristoffer Sandås, Leticia Spindola, Solveig Løkhammer, Anne-Kristin Stavrum, Ole Andreassen, Markos Tesfaye, Stéphanie Le Hellard

**Affiliations:** 1 Department of Clinical Science, University of Bergen, Bergen, Norway; 2 Dr. Einar Martens Research Group for Biological Psychiatry, Department of Medical Genetics, Haukeland University Hospital, Bergen, Norway; 3 Bergen Center for Brain Plasticity, Haukeland University Hospital, Bergen, Norway; 4 School of Bioscience, University of Skövde, Hjälmgatan 14, Skövde, Tidaholm 52236 Sweden; 5 Center for Precision Psychiatry, Division of Mental Health and Addiction, Oslo University Hospital, and Institute of Clinical Medicine, University of Oslo, Oslo, Norway

**Keywords:** Methylation scores, Schizophrenia, Methylome-wide association studies, Summary statistics, LDpred2, Polygenic risk score, MRS, EWAS

## Abstract

**Objective:**

This study sought to determine if the R package LDpred2, designed for polygenic risk score creation for genome-wide association studies using summary statistics, could be adapted for deriving DNA methylation scores from methylome-wide association studies. Recognizing that linkage disequilibrium, used as prior in LDpred2, does not apply to methylation, we explored co-methylated regions and topologically associating domains as alternative structural priors for correlation between methylation sites. A genomic sliding-window approach was also tested. The performance of the LDpred2-based models was evaluated on methylation data from schizophrenia and control samples (*N* = 1,227).

**Results:**

LDpred2 models employing topologically associating domains and sliding window clusters as priors performed similarly to existing methods, explaining approximately 3.6% of schizophrenia phenotypic variance. The co-methylated regions model underperformed due to insufficient clustering of probes. The similarity in performance between the model using topologically associating domains and a null model consisting of random clusters suggests that the structural information provided by these domains enhances performance only marginally. In conclusion, while LDpred2 can be adapted for methylation data, it does not substantially enhance methylation score performance over existing methods, and the choice of structural prior may not be a critical factor.

## Introduction

DNA methylation can be analyzed on a large scale in methylome-wide association studies (MWAS), which measure the statistical association between methylation levels and a phenotype, similar to the process of genome-wide association studies (GWAS) [1, 2]. Methylation scores (MS) are constructed from MWAS results, analogous to polygenic risk scores (PRS) in GWAS. This creates a single score as a way of interpreting the association study.

MS have shown promise in the study of complex mental disorders such as schizophrenia [3]. We recently showed that 3.5% of schizophrenia phenotypic variability could be explained by methylation using a pruning and thresholding (P + T) method for computing MS [4]. However, there are several advanced methods, developed for PRS, outperforming P + T, that have yet to be tested for MS [5–7]. Many of these methods use summary statistics, which enable pooling of data from multiple studies, and employ techniques such as Bayesian regression, regularized linear regression, and Markov chain Monte Carlo algorithms. One of the most popular and best-performing implementations of such a method is the R package LDpred2, which utilizes a Gibbs sampler using external reference linkage disequilibrium (LD) correlation maps as prior [8, 9].

Here, we aimed to adapt LDpred2 for methylation data and compare the resulting MS with the hitherto best-performing P + T method mentioned above. To this end, the study comprised the following objectives:

I) Finding a suitable substitute for LD as a prior, since LD does not structurally characterize methylation. Co-methylated regions (CMRs) and topologically associating domains (TADs) were tested as priors [10, 11].
II) Creating MS using these priors with LDpred2 from schizophrenia MWAS summary statistics [4], to facilitate comparison with the P + T method.
III) Evaluating the results using the same process as Tesfaye et al. [4], by regressing the MS against the known phenotypes, obtaining a pseudo R^2^-value for the model.

## Main text

### Data

The training data (*N* = 2,015) for the LDpred2 MS models was obtained from the EWAS meta-analysis of schizophrenia described in our previous publication [4]. Pre-processed individual-level peripheral blood methylation data from schizophrenia and control samples from the NORMENT TOP sample was used as test data (*N* = 1,227) [12]. The test data methylation betas have been residualized, removing the effects of the covariates age, smoking scores, estimated cell-type proportions, batch effects, genotype principal components, methylation principal components, and surrogate variables [4].

### Methods

The following R packages were used: CoMeBack version 0.1.0, bigsnpr version 1.12.2, which includes bigstatsr and LDpred2 [10, 13].

#### LDpred2

LDpred2 uses a Gibbs sampler with a Bayesian approach, where posterior effect sizes are calculated from GWAS summary statistics and an LD prior [5]. The LDpred2 workflow allows for use of a pre-specified set of LD blocks from an external reference, instead of the standard sliding-window approach. A critical aspect of this method is the use of a probe-probe pairwise sparse correlation matrix.

#### CoMeBack

CoMeBack calculates CMRs and assigns probes based on genomic proximity and correlation in methylation levels from a test set [10]. The package also considers the number of known methylation sites between the probes to define a CMR. Important settings in CoMeBack are *corlo*: the probe-probe correlation threshold, *maxprbdst*: the maximum distance between probes, and *corlodst*: the maximum distance between inter-probe CpGs.

### Implementation

The models were created using the LDpred2-auto function, which infers the hyperparameters *h*^*2*^ and *p* internally and does not require a validation set [5]. Correlation matrices were created by clustering the probes in the test set using the processes described below, and by calculating the pairwise probe-probe Pearson correlations in methylation levels within each cluster or block. Only probes common to both the training and the test set were used in the analysis.

### Co-methylated regions

CoMeBack was used to create CMR clusters with the following lenient settings, to allow for as many probes as possible to cluster, while keeping the thresholds reasonable: *corlo* = 0.2, *maxprbdst* = 100,000 bp, and *corlodst* = 800 bp. These clusters were used as blocks in the correlation matrix. A second matrix was created using only the non-singleton clusters from this analysis, i.e. clusters containing two or more probes. The *h*^*2*^-value produced in the pipeline was negative for the non-singleton matrix, which is not allowed in the Gibbs sampler. Therefore, the *h*^*2*^-value was set to 10^− 5^ in this analysis before proceeding.

### Sliding window approach

The CoMeBack pipeline was modified to only consider genomic proximity, by setting *corlo* = 10^− 10^, effectively creating a sliding window process which clusters probes on each chromosome. Bypassing the correlation element is not a major issue since the correlations are taken into account in the LDpred2 Gibbs sampler when calculating the posterior effect sizes [5]. Six versions were created with window sizes 5 kilobases (kb), 10 kb, 20 kb, 100 kb, 500 kb and 1 megabase (Mb). Both *maxprbdst* and *corlodst* were set to the respective window size in each analysis. Correlation matrices were produced using these clusters as blocks.

### Topologically associating domains (TAD)

The TAD scaffold created by Rohit & Bonnie [14] was acquired on March 15, 2024, and converted to genome build hg19 using USCS Liftover with standard settings [15]. The scaffold contains consensus start and end positions of TADs in each chromosome, calculated by combining information from seven different human cell types [14]. Since the test data comes from blood samples, which contain several cell types, a consensus scaffold was chosen rather than a cell-specific one. The rationale behind this was that a general mapping would be more robust compared to a cell-specific mapping, since the exact cell composition in the samples are not known. The probes in the test set were then mapped to the TADs using their genomic locations obtained from the Illumina 450k and EPIC manifests included in the CoMeBack package, and the resulting 2880 clusters were used as blocks in the matrix.

### Random clusters

To assess the results from the TAD clusters and determine if the structural information in the TADs contributed to the model, ten sets of random clusters were created and used for matrix blocks as a null model. The observed distribution of the number of probes in the 2,880 TAD clusters was found to be characterized by the following statistics: min = 1, Q_1_ = 45, median = 82, mean = 118, Q_3_ = 138, and max = 2,690. This suggests a log-normal distribution, so to roughly correspond to the observed distribution in the TAD clusters, the number of probes (*P*) in the random clusters was drawn from the distribution *P* ~ LogNorm(mean = log(82), SD = 0.7). The number of clusters (*C*) in each random set was drawn from the distribution *C* ~ Norm(mean = 2880, SD = 144), to ensure that each random set contained roughly the same number of clusters as the TAD set. *P* probes were then randomly assigned to the *C* clusters in each set.

### Applying and evaluating scores

The scores were calculated by the multiplication **S** = **M**^**T**^**β**, where **S** is the scores, **M**_**(probes x samples)**_ is the methylation levels from the test set and **β** is the posterior effect sizes from the LDpred2 Gibbs sampler. The scores were then evaluated by performing logistic regression of the scores against the known schizophrenia phenotypes for the samples in **M**, following the procedures described previously [4]. A Nagelkerke R^2^-value was calculated from the regression [16]. P-values and AIC values from the logistic regression were also included in the evaluation.

## Results & discussion

Two priors were tested with LDpred2 for creating MS: CMRs and TADs. Two models were created using the CMR prior: one including singleton clusters, and one excluding them. Additionally, sliding window models were created with CoMeBack using six different window sizes (5 kb, 10 kb, 20 kb, 100 kb, 500 kb and 1 Mb). Ten sets of random clusters were created as a null model for the TAD-based clusters. The models were compared to the existing P + T model using logistic regression. The performances of the TAD-based model, the sliding window models, and the random cluster models were almost identical to the P + T model (Table 1).

**Table 1:** Main results of the model comparisons

Model	*p*-value	Nagelkerke *R*^2^	AIC	Probes clustered*
P + T (Tesfaye et al. 2024)	3.49 × 10^− 8^	0.0347	1597.7	12%
LDpred2, CMR blocks	0.432	0.000687	1628.8	18%
LDpred2, CMR blocks, no singletons	8.39 × 10^− 7^	0.0275	1604.3	18%
LDpred2, CMR sliding window blocks**	[1.83 to 1.88] × 10^− 8^	0.0361 to 0.0362	1596.3 to 1596.5	74–86%
LDpred2, TAD blocks	1.68 × 10^− 8^	0.0363	1596.2	99%
LDpred2, random cluster blocks	[2.02 to 2.79] × 10^− 8^	0.0352 to 0.0359	1596.6 to 1597.2	99%

* The percentage of probes included in non-singleton clusters. In the model *LDpred2*,* CMR blocks*,* no singletons*, this means that only 18% of the probes were used in the analysis, since singleton clusters were discarded

** For detailed results, see Appendix II

CMR: co-methylated region, TAD: topologically associating domain

The CMR-based model which included singleton clusters performed poorly, due to creating only a partial block structure in the correlation matrix (Appendix I). When removing the singleton clusters, the model performed substantially better, but the negative *h*^*2*^-value indicated that the correlation matrix likely had negative eigenvalues, most probably due to ill-conditioning, and therefore the model was discarded. The proportion of non-singleton clusters was too low in the CMR models to function properly with the algorithm, despite the relatively lenient settings used in CoMeBack.

The TAD-based model performed the best by a small margin, supporting the hypothesis that using TADs as a structural prior contributes valuable data to methylation models. The null model, consisting of ten sets of random clusters with approximately the same number of clusters and probes per cluster as the TAD model, however, showed nearly identical results as the TAD model. This suggests that it is the algorithm in itself, and its use of probe-probe correlation in the posterior effect sizes, that led to improved performance results, and not the TAD structures (5).

The sliding window model results were also nearly identical to the TAD results, suggesting that a perfect clustering of all probes is unnecessary. Even the 5 kb sliding window, which only clustered 74% of probes into non-singleton clusters, performed as well as the TAD clusters, and the much smaller cluster sizes in the sliding window versions did not seem to affect the model negatively (Appendix II). This offers some support for using sliding window approaches based solely on genomic location in MS creation if, as in this case, correlations in methylation levels are processed in some other step in the analysis.

An important difference between PRS and MS creation is the imputation of probes between microarray typing and analysis in GWAS. Following genotyping of SNP arrays with a limited number of SNPs, LD references are used to impute SNPs in between probes, increasing the number of SNPs in the analysis from 400,000 to about 11,000,000 [17]. When creating PRS, these SNPs are removed again by pruning to reduce redundancy. There is no similar imputation and pruning in MWAS. This difference suggests that pruning according to a structural reference, for example TADs or CMRs, might not be as crucial in MS creation as LD pruning is in PRS. The performances of the sliding-window models and the earlier P + T model suggests that removing redundancy by considering probe-probe correlation is sufficient.

In conclusion, the LDpred2-based models performed marginally better than the existing, more validated P + T model, showing that it is feasible to use PRS methods for MS. Furthermore, the results from the LDpred2-based models support the findings from our previous study, that about 3.5% of the schizophrenia phenotypic variance can be explained by DNA methylation using MS [4]. The CMR priors functioned poorly in the analysis, and the TAD prior did not outperform the random cluster null model, suggesting that the key step in the process for MS creation is limiting the number of probes included by pruning, or in the case of LDpred2, regularization [5].

Both LDpred2 and CoMeBack, the latter also used in the pruning step for the P + T method [4], rely heavily on pairwise correlation. This merits a future investigation into other types of algorithms, such as random forests or neural networks, that do not depend on standard linear statistical methods.

### Limitations

- The LDpred2 models were only tested on a single dataset.
- The models were only tested on a single phenotype.
- The blocks in the matrix were created using the test set, risking potential data leakage. Ideally, block matrices would have been created using a large, independent data set, just as has been done for LD in PRS [5].
- The LDpred2-auto function used for the models does not require a validation set to tune the hyperparameters, and is reported by Prive et al. [5] to perform nearly as well as the -grid version. Optimally, however, the tests would have been performed with an independent validation set and the -grid model.
- The sample sizes for training MWAS and test sample were 2,015 and 1,227, respectively, which might limit the power of the MS. Further tests might be needed on larger samples.

## Appendix I

**Fig. 1:**
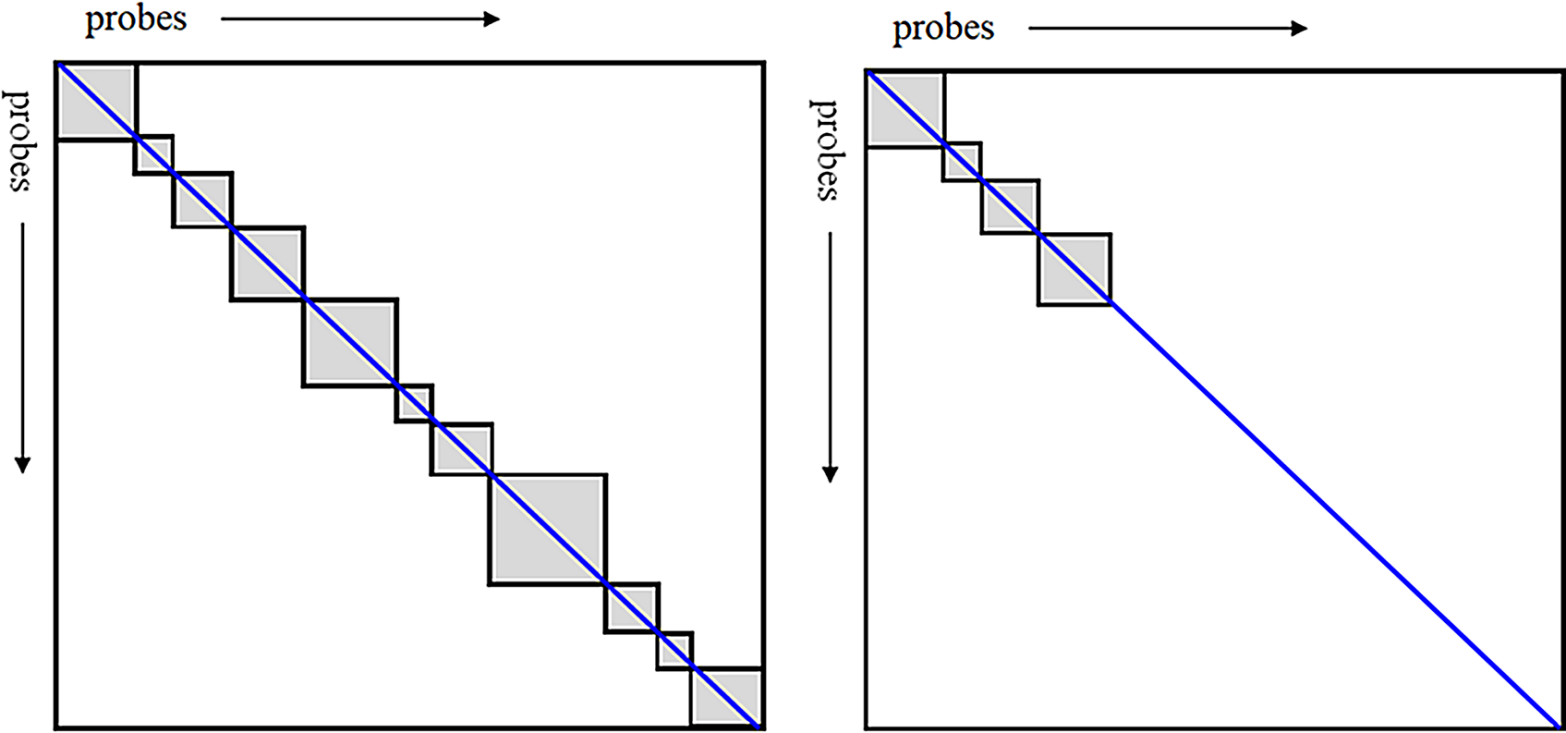
Left: Schematic of a probe-probe block correlation matrix. Grey areas represent blocks around the diagonal, within which correlations are calculated. Most fields are empty (white). Right: When including singletons in the CMR blocks, only a partial block structure is created, which gives the algorithm too little correlation information to work with, since the singleton clusters do not contribute anything to the model

## Appendix II

**Table 2:** Distributions of probes per cluster in the TAD and sliding window clusters

Window size	Probes clustered*	Min	Q1	Q2	Mean	Q3	Max	*p*-value	Nagel- kerke R^2^	AIC
5kb	74%	1	1	2	2.31	3	91	1.88 × 10^− 8^	0.0361	1596.5
10kb	78%	1	1	2	2.50	3	119	1.83 × 10^− 8^	0.0361	1596.4
20kb	81%	1	1	2	2.71	3	119	1.85 × 10^− 8^	0.0362	1596.3
100kb	85%	1	1	2	3.03	4	119	1.85 × 10^− 8^	0.0362	1596.3
500kb	86%	1	1	2	3.09	4	119	1.84 × 10^− 8^	0.0361	1596.3
1Mb	86%	1	1	2	3.09	4	119	1.86 ×10^− 8^	0.0361	1596.3
**TAD clusters**	**99.99%**	**1**	**45**	**82**	**118.46**	**138**	**2690**	**1.68 × 10** ^**− 8**^	**0.0363**	**1596.2**

^*^The percentage of probes included in non-singleton clusters

TAD: topologically associating domain

The increase in probes clustered diminishes with larger window sizes and the percentage is constant above 500kb. The distributions change very little after 10kb. The differences in probe distributions between the sliding window and TAD clusters did not have any significant effect on the methylation scores’ performance

## Data Availability

The training data used in this study is freely available at https://figshare.com/articles/dataset/SCZ_MWAS_meta_analysis_GSE80417_GSE152027_GSE84727_csv/28466084?file=52549541. This dataset is a meta-analysis of the datasets GSE80417, GSE152027, and GSE84727, downloadable from Gene Expression Omnibus https://www.ncbi.nlm.nih.gov/geo/. Details about the meta-analysis can be found in our previous publication (4). The test data is not available due to privacy concerns, as it contains individual patient data from NORMENT. The code used in the analysis is available at https://github.com/KristofferSandas/methylation_ldpred2.

## Abbreviations

CMR: Co-methylated region
GWAS: Genome-wide association study
Kb: Kilobase
LD: Linkage disequilibrium
Mb: Megabase
MS: Methylation score
MWAS: Methylome-wide association study
NORMENT: Norwegian Center for Mental Disorders Research
PRS: Polygenic risk score
P + T: Pruning and thresholding
SNP: Single-nucleotide polymorphism
TAD: Topologically associating domain
TOP: Thematically Organized Psychosis
